# Obtaining Active Polylactide (PLA) and Polyhydroxybutyrate (PHB) Blends Based Bionanocomposites Modified with Graphene Oxide and Supercritical Carbon Dioxide (scCO_2_)-Assisted Cinnamaldehyde: Effect on Thermal-Mechanical, Disintegration and Mass Transport Properties

**DOI:** 10.3390/polym13223968

**Published:** 2021-11-17

**Authors:** Carolina Villegas, Alejandra Torres, Julio Bruna, María Ignacia Bustos, Alvaro Díaz-Barrera, Julio Romero, Adrián Rojas, Abel Guarda

**Affiliations:** 1 Center for Packaging Innovation (LABEN), Center for the Development of Nanoscience and Nanotechnology (CEDENNA), Technology Faculty, University of Santiago de Chile (USACH), Santiago 9170201, Chile; carolina.villegasv@usach.cl (C.V.); julio.bruna@usach.cl (J.B.); maria.bustos.c@usach.cl (M.I.B.); adrian.rojass@usach.cl (A.R.); abel.guarda@usach.cl (A.G.); 2 Escuela de Ingeniería Bioquímica, Pontificia Universidad Católica de Valparaíso, Valparaíso 2340000, Chile; alvaro.diaz@pucv.cl; 3 Laboratory of Membrane Separation Processes (LabProSeM), Department of Chemical Engineering, Engineering Faculty, University of Santiago de Chile, Santiago 9170201, Chile; julio.romero@usach.cl

**Keywords:** PLA/PHB blend, bionanocomposites, graphene oxide, nano-reinforcement, release kinetic

## Abstract

Bionanocomposites based on Polylactide (PLA) and Polyhydroxybutyrate (PHB) blends were successfully obtained through a combined extrusion and impregnation process using supercritical CO_2_ (scCO_2_). Graphene oxide (GO) and cinnamaldehyde (Ci) were incorporated into the blends as nano-reinforcement and an active compound, respectively, separately, and simultaneously. From the results, cinnamaldehyde quantification values varied between 5.7% and 6.1% (*w*/*w*). When GO and Ci were incorporated, elongation percentage increased up to 16%, and, therefore, the mechanical properties were improved, with respect to neat PLA. The results indicated that the Ci diffusion through the blends and bionanocomposites was influenced by the nano-reinforcing incorporation. The disintegration capacity of the developed materials decreased with the incorporation of GO and PHB, up to 14 and 23 days of testing, respectively, without compromising the biodegradability characteristics of the final material.

## 1. Introduction

The use of plastic materials derived from petroleum provides multiple advantages and facilities in human life, being used in different areas, such as medicine, agriculture, and food packaging, among others. However, this type of material has generated a high environmental impact, due to its slow degradation [1]. Unfortunately, they are accumulating, occupying a significant volume in landfills and oceans today. For these reasons, biodegradable materials from renewable sources are a very important option in achieving the goals of sustainable development and reducing negative environmental impact. The main advantages of biodegradable materials from renewable sources are their biodegradation in a biologically active environment and their contribution to lowering carbon footprint [2]. Among these materials are biopolyesters, including polyhydroxybutyrate (PHB) and polylactide (PLA). These polymers are polyesters that are synthesized using bio-resources such as sugar or plant oil and are characterized by good biocompatibility, biodegradability, and sustainability, which make them a potential alternative for biomedical applications [3,4,5,6]. Biopolyesters can be modified via polymer–polymer blending, some of the commonly employed techniques include copolymerization, crosslinking, composition, and blends [7]. Polymer blending is a convenient physical modification technique to blend the properties of different polymers or to generate novel properties. Blending eliminates the requirement to develop new polymers or copolymers and can tailor a material with the desired properties via a thermodynamically driven mixing of two or more polymers [7]. In this way, PLA can be blended with polymers and biopolymers [8,9]. Separately, PLA and PHB are characterized by poor processing [10], nevertheless, blending PLA with PHB, which is a highly crystalline biopolyester, results in new materials with interesting physical, thermal, and mechanical properties in comparison to neat PLA [11]. Zhang and coworkers (2011) investigated the morphology, structure, crystallization, thermal properties, mechanical properties, and biodegradation of PLA/PHB blends, which were prepared by melt mixing. The PLA/PHB blend in 75/25 ratio showed improved mechanical properties, thermal decomposition temperature, and the presence of some interactions [12]. Meanwhile, Ausejo and coworkers (2018) investigated the PLA/PHB blends for their improved utility properties and biodegradability for use in additive manufacturing-3D printing [13]. Besides, PLA, PHB, and their blends can be considered for important biomedical applications as the production of conventional medical implantation devices, tissues engineered and controlled drug delivery systems, including repair patches, drug delivery platforms, wound healing, and biocompatible sutures [6,14,15,16].

However, these biopolyesters or their blends may present certain limitations in terms of their mechanical, barrier properties, or lack of intrinsic bioactivity due to the absence of antibacterial behavior. Therefore, one of the ways to increase these properties is by incorporating active compounds and different types of fillers into the polymeric matrix, thus improving properties such as elasticity and permeability to gases such as water vapor and oxygen [17]. Different routes have been reported to overcome these issues where the addition of particles into the matrix to produce composite materials is one of the most used. Different kinds of particles had been used such as fibers, nanocrystals, and micro or nanoparticles [18,19,20]. Luzi and coworkers (2019) developed biodegradable multicomponent films based on PLA/PHB plasticized with oligomeric lactic acid (OLA), reinforced with synthetized cellulose nanocrystals (CNC), and modified by a natural additive with antimicrobial activity (carvacrol). The films were formulated and processed by extrusion process. Morphological, mechanical, and thermal properties were tested to determine the effect of different components in comparison with neat PLA. Results showed the positive effect of CNC with the increase of Young’s modulus associated with an increase in the elongation at break [20]. Moreover, the mechanical resistance of these biopolymers has been improved by the incorporation of nanofillers—such as organically modified nanoclay (OMMT) and/or modified montmorillonite Cloisite 30B (C30B)—produced a reinforcing effect into PLA-PHB matrices owing to the enhancement of the biopolymers’ interfacial adhesion in the blend [21].

Other types of nano-reinforcements used in recent years are the graphene derivatives including graphene nanoplatelets, exfoliated graphite, or graphene oxide, which have emerged as a potential alternative due to their exciting properties recently [22]. Graphene oxide (GO) is a unique material; it is a monomolecular layer of graphite with various oxygen-containing functionalities such as epoxide, carbonyl, carboxyl, and hydroxyl groups. Graphene-based materials possess high surface area, excellent electrical conductivity, high mechanical strength, supreme thermal conductivity, and good biocompatibility [23]. Recent studies have also established that graphene-based materials show excellent antibacterial and angiogenic properties [24]. Graphene and its derivatives are used in three forms: the pristine form; mixed with other antibacterial agents, such as Ag, chitosan, active compounds; or with a base material, such as poly (N-vinylcarbazole) (PVK) and poly (lactic acid) (PLA) [25]. Davoodi and coworkers (2018) produced PLA-GO nanocomposite nanofibers via electrospinning and study the location and arrangement of GO sheets in the obtained mats by atomic forced microscopy (AFM) and scanning electron microscopy (SEM). They showed that the localization of nanoparticles by different methods controls different final properties. Besides, observed an improvement in mechanical properties indicated that parts of GO sheets with small lateral size (less than 300 nm) can be placed inside of the electrospun fibers [26]. Further, have been developed nanocomposite thin films of poly (lactic acid) (PLA) incorporating small amounts (0.2 to 1 wt%) of graphene oxide (GO) and graphene nanoplatelets (GNP). The films improved in tensile strength and Young’s modulus was about 15 and 85%, respectively. Permeabilities towards oxygen and nitrogen decreased, respectively, three and fourfold in a film loaded with both GO and GNP [27].

On the other hand, cinnamaldehyde (Ci) is a biologically active compound present in the essential oil of the genus *Cinnamomum*, recognized as a strong antioxidant and antimicrobial agent [28] and it is currently used in the pharmaceutical, cosmetic, and food industries. There are numerous techniques to incorporate active compounds in polymer matrices, such as the solvent casting method [29], mainly used on a laboratory scale, coating [30], electrospinning, and melt extrusion process [31]. Despite being relatively simple techniques, many drawbacks have been observed at an industrial scale, including the use of toxic organic solvents that must be removed from the matrices, unwanted reactions between substances, such as the possible volatilization or thermal degradation of the active agent. In this way, the incorporation of these compounds into polymeric matrices by means of the supercritical impregnation process has been reported in the last years as an efficient alternative for this purpose [28,32,33,34,35,36,37,38,39,40,41,42].

To the best of the authors’ knowledge, and from the above literature review, no work has been carried out on the development of active PLA/PHB-based bionanocomposites modified with Graphene Oxide and scCO_2_-assisted Cinnamaldehyde. This work proposes the obtaining of bionanocomposites through an extrusion process widely used in the industrial area combined with supercritical impregnation, a green process free of organic solvents, and that avoids thermal degradation and therefore, loss of functionality of active compounds. In this way, the aim of this work is to evaluate the effect of the incorporation of both the nano-reinforcement GO and the impregnated Ci active compound on the thermal-mechanical, disintegration, and mass transport properties of PLA/PHB blends and bionanocomposites, which could have potential biomedical applications.

## 2. Materials and Methods

### 2.1. Reagents and Strains

Poly (lactic acid) (PLA), 2003D (specific gravity 1.24; MFR g/10 min (210 °C, 2.16 kg)), was purchased from Natureworks^®^ Co. (Minnetonka, MN, USA). Graphene Oxide (GO) (Av-GOx-40, lateral size 40 µm, specific surface area of 400 m^2^ g^−1^, thickness 1–2 nm, oxygen content 30%) was purchased from Avanzare^®^ (La Rioja, España).

### 2.2. Microorganisms, Culture Medium, and Obtained PHB

Poly (3-hydroxybutyrate) (PHB) was produced using the *Azotobacter vinelandii* ATCC 9046 *(wild-type strain)*. The bacteria will grow in a medium of the following composition (g/L): 20 sucrose, 0.66 K_2_HPO_4_, 0.16 KH_2_PO_4_, 0.05 CaSO_4_, 0.2 NaCl, 0.2 MgSO_4_·7H_2_O, 0.0029 Na_2_MoO_4_·2H_2_O, 0.027 FeSO_4_ purchased from Aldrich^®^ Chemistry (St. Louis, MO, USA). The inoculum for the bioreactor (Applikon, Schiedam, Netherlands) of 3 L will be prepared in 500 mL Erlenmeyer flasks, containing 100 mL of culture medium at 200 rpm and 30 °C in an orbital incubator shaker (Daihan LabTech CO, Namyangju, Korea).

The PHB production will be performed using a 3 L bioreactor operated in batch-mode. The working volume was 2 L, at 30 °C and pH 7.0 was controlled by automatic addition of 2 M NaOH from purchased from Aldrich^®^ Chemistry (St. Louis, MO, USA). The dissolved oxygen tension (DOT) will be measured using a polarographic oxygen probe (Ingold, Mettler-Toledo Inc., Columbus, OH, USA). The batch cultures were conducted at 300 rpm [41]. PHB extractions from the cells were carried out using chloroform and sodium hypochlorite from Merck (Darmstadt, Germany), according to García and coworkers (2019) [42].

### 2.3. Preparation of Blends and Bionanocomposites

#### 2.3.1. PLA/GO Masterbatch

The masterbatch method was used in order to homogenize the GO nano-reinforcement. PLA/GO pellet with a 5% *w*/*w* concentration of GO was processed into a co-rotating twin-screw extruder LabTech Scientific (LTE20 model) (Samutprakarn, Thailand). The temperature profiles from zone 1 to zone 5 were kept between 160 and 180 °C. The twin-screw speed was fixed at 80 rpm and the single screw hopper feeder was fixed at 50 rpm. The masterbatch extruded were collected in a Bench Top Chill Roll Line Type LBCR-150 (Samutprakarn, Thailand), the cutting speed was 9.5 m/min, and the pressure was 25 bar.

#### 2.3.2. Extrusion of Blends and Bionanocomposites

First, PLA powder was previously dried at 80 °C for 24 h. The PLA films, PLA/PHB blends (75:25% *w*/*w*), and PLA/PHB/GO bionanocomposites (the latter obtained with 0.5% *w*/*w* of the masterbatch explained in Section 2.3.1) were obtained through the melt extrusion process. The temperature used ranged from 190 to 198 °C (zone 1 to zone 5) with a die temperature of 200 °C. The screw speed and the feed were fixed at 12.5 rpm; the different materials were collected in a Scientific Labtech LBCR-150 chill roll attachment (Samutprakarn, Thailand) at 2.2 rpm.

### 2.4. Supercritical Impregnation of Cinnamaldehyde (Ci) in Blends and Bionanocomposites

Supercritical impregnations of Ci in the samples were performed using the apparatus schematically described in Torres and coworkers [34]. Briefly, the process was carried out into a 100 mL high-pressure cell, where Ci (0.5 mL) was placed at the bottom of a vessel in a glass container. The blends and bionanocomposites (200 cm^2^, 103.2 ± 3.2 μm average thickness) were separated by a metal support and placed into the cell. CO_2_ was loaded into the system by means of an ISCO 500D syringe pump (Lincoln, NE, USA) operated at a constant pressure rate during the impregnation runs. The experiments were carried out to 12 MPa of pressure and 1 MPa min^−1^ of depressurization rates at a constant temperature of 40 °C for 3 h. The conditions for the supercritical impregnation experiments were selected according to previous results reported by the same research group [28,37]. The temperature of the high-pressure cell was controlled using a thermostatic electric resistance around the cell.

### 2.5. Characterization of Graphene Oxide (GO), Blends and Bionanocomposites

#### 2.5.1. Quantification of Cinnamaldehyde in Blends and Bionanocomposites

In order to study the effect of the Ci incorporation, the active compound effective concentration in PLA, PLA/PHB, and PLA/PHB/GO were determined after each supercritical impregnation process. The analysis was performed using a method of dissolution and precipitation of the modified polymer [43], followed by detection and quantification of the active compound carried out through high-performance liquid chromatography (HPLC). The procedure was carried out according to the explanation by Villegas and coworkers [28]. All results were expressed as % Ci with respect to polymer weight. The analyses were performed in triplicate.

#### 2.5.2. Attenuated Total Reflectance Fourier Transforms Infrared (ATR-FTIR) Spectroscopy

The different functional groups’ identification and interaction were carried out for the different samples. FTIR spectra of developed films were recorded using a Bruker Alpha spectrometer (Wismar, Germany) equipped with an attenuated total reflection diamond crystal accessory (Bruker^®^ Platinium, Billerica, MA, USA) and analysis was performed using OPUS^®^ Software Version 7 (Bruker^®^ Platinium, Billerica, MA, USA). The spectra were obtained with a resolution of 4 cm^−1^ in a wavenumber range from 4000 to 400 cm^−1^ with 24 scans per sample.

#### 2.5.3. Thermal Properties

The assays were carried out by means of Differential Scanning Calorimetry using a Mettler Toledo DSC model 822e (Schwarzenbach, Switzerland) over the 0 °C to 300 °C temperature range. The heating scan of the samples was done at 10 °C min^−1^ and 8–10 of sample mg were placed in hermetically sealed capsules.

On the other hand, the thermal stability of the samples was tested with a Mettler Toledo Gas Controller GC20 Stare System TGA/DCS (Schwarzenbach, Switzerland). Approximately 7 mg of the sample was spread uniformly on the bottom side of the alumina crucible (70 μL) and was heated from 30 to 600 °C at 10 °C min^−1^. All experiments were carried out under a nitrogen atmosphere. The analyses were performed in triplicate.

#### 2.5.4. Mechanical Properties

Elongation at break, modulus of elasticity, and tensile strength of each material was measured using a Zwick Roell model BDOFB 0.5 TH Tensile Tester (Ulm, Germany), according to ASTM D-882 [44]. Tests were performed in rectangular probes (10 cm × 2.5 cm) at 23 °C and 50% relative humidity (RH) for 48 h before the test. Results are the average of 20 specimens for each film. The analyses were performed in triplicate and the results were expressed as the average value ± standard deviation.

### 2.6. Disintegration under Composting Conditions

The objective was to evaluate the effect of GO and Ci on the disintegration process of PLA and PLA/PHB blend under composting conditions as the sustainable end-of-life option. The analysis was carried out according to the procedure explained by Villegas and coworkers [37], with few modifications, following the ISO-20200 standard [45].

For this purpose, samples (2.5 cm × 2.5 cm) were prepared, and they were buried at a 6 cm depth in plastic reactors containing the solid synthetic wet waste [46]. Each sample was contained in a textile mesh to allow their easy removal after treatment but allowing the access of microorganisms and moisture [47]. In this case, the extraction of the disintegrated samples was carried out from the day after the start of the process (day 0) during defined time intervals (1, 3, 7, 9, 14, 17, and 23 days). These extractions were carried out carefully, cleaning each textile mesh and removing the film inside each one. Subsequently, the films were dried for 10 min at 105 °C and then at 40 °C for 24 h. From this, it was possible to make a photographic record and obtain the weight. The analyses were performed in duplicate.

Photographs with digital camera (Canon IXUS 195. Amstelveen, Netherlands) were taken to all samples once extracts from the composting medium for visual comparison. On the other hand, the disintegration degree was calculated by normalizing the sample weight at each day of incubation to the initial weight. In order to determine the time at which 50% of each film was degraded, disintegrability degree values were fitted using the Boltzmann equation [48].

### 2.7. Kinetic Release of Active Compound from PLA/PHB Blends and Bionanocomposites

The Ci release kinetic study and mass transfer properties determination is focused on the final disposition and the applicability of the blends and bionanocomposites. It is also important to establish the concentration of Ci over time and the affinity it has in different mediums. The release of the active compound from films and blends with and without GO was carried out into 10% *v*/*v* and 50% *v*/*v* ethanolic solutions, according to European regulation [49] following the procedure described by Torres and coworkers (2014) [32]. Release experiments were conducted with the aim to study the transport and thermodynamic properties of the active compound in the developed materials. Impregnated film and blends samples with 50 cm^2^ surface area were totally immersed into glass tubes filled with 130 mL of ethanol solutions. Films were placed in the tubes using metal supports to avoid direct contact between samples and with the wall of the tube. These tubes were stored at 40 °C in an oven for at least 10 days. Ethanol simulant samples of 1 mL were collected from the tubes as a time function. These samples were analyzed by means of HPLC (see Section 2.5.1) to determine the concentration of release Ci as a time function. The analyses were performed in duplicate.

#### 2.7.1. Mathematical Modeling of Active Release Kinetic from Impregnated Blends and Bionanocomposites

The release kinetic of Ci from different materials can be explained by means of a diffusion process through the polymeric material coupled to the convective transport in the receiving phase if this phase is a fluid. Thus, Ci transfer from the blends and bionanocomposites to the solutions during the release process can be described by means of Fick’s Law. This approach based on classical phenomenological equations and thermodynamic considerations has been previously proposed in the literature by the same research group [32,34,36,50].

In this way, a few assumptions had to be made in order to use the model:

(1) The initial Ci concentration in the different materials is known and is homogeneously distributed in the polymer.
(2) The ethanolic solutions are initially Ci free.
(3) There is no chemical interaction between the simulant solutions and polymer (not swelling).
(4) No mass transfer limitation in the release medium, so the Ci is homogeneously distributed in the bulk of them.

From this, mass transfer equations were solved under steady-state conditions for an instantaneous time. Regula Falsi method was applied in order to reduce the number of interfacial concentration values of cinnamaldehyde iterations. Taking into account that the initial concentration of cinnamaldehyde in the polymer and the solution bulk are known, an iterative calculation can be done to estimate the mass transfer flux from the polymer to simulants. The mathematical model was used to simulate different release kinetics using different values of the effective diffusion coefficient of Ci in the polymers, D_eff_ [38]. The chosen D_eff_ value is the one that shows the closest values of simulated and experimental release kinetics. These calculations were implemented by means of a program built in Matlab 7.1 (MathWorks Inc., Natick, MA, USA).

### 2.8. Statistical Analysis

A randomized experimental design was considered for the experiments. Data analysis was carried out using Statgraphics Plus 5.1 (StatPoint Technologies, Warrenton, VA, USA). This software was used to implement variance analysis and Fisher’s LSD test. Differences were considered significant at *p <* 0.05.

## 3. Results and Discussion

### 3.1. Characterization of Active Impregnated Blends and Bionanocomposites

#### 3.1.1. Supercritical Impregnation of Cinnamaldehyde in Blends and Bionanocomposites

**Figure 1:**
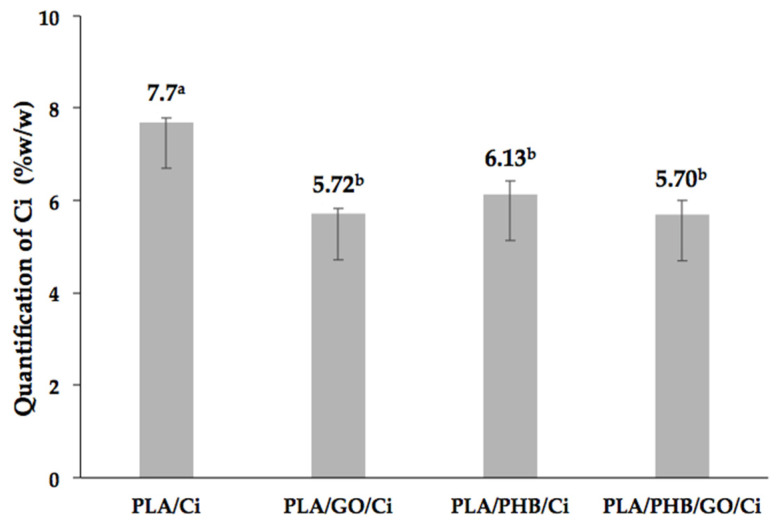
Quantification of impregnated cinnamaldehyde (% *w*/*w*) in processed films and blends at process conditions of 12 MPa pressure and 1 MPa min^−1^ depressurization. Mean value (*n* = 3). Parameters in bar chart denoted with the same letters (a–b) do not differ statistically at the level of confidence 0.05.

Figure 1 shows the results obtained for the effective amount of Ci incorporated after the scCO_2_ impregnation process in PLA, PLA/GO, PLA/PHB, and PLA/PHB/GO films and blends. From the results, it can be appreciated in the higher incorporation of the active compound in the PLA film while for the other samples the percentage was maintained without observing significant differences between them.

From the figure, it can be seen that the GO incorporation in the blends decreases the effective incorporation of the active compound from 6.13 to 5.70 (% *w*/*w*). This behavior could be due to the chemical interaction between the polymer chains and the GO sheets, which would make it difficult to incorporate the cinnamaldehyde. Polar groups of PLA as the C=O allow it to interact with the –OH of GO through hydrogen bonding [51], this could generate a saturation of the polar binding sites. In addition, the structure of this nano-reinforcement is on the order of 1 µm lateral, a thickness of approximately 1 nm [51,52,53], and has a theoretical specific surface area of up to ~2600 (m^2^ g^−1^) [54]. These characteristics would make it difficult to incorporate Ci into the matrix, due to steric hindrance [55].

#### 3.1.2. Attenuated Total Reflectance Fourier Transforms Infrared (ATR-FTIR) Spectroscopy

**Figure 2:**
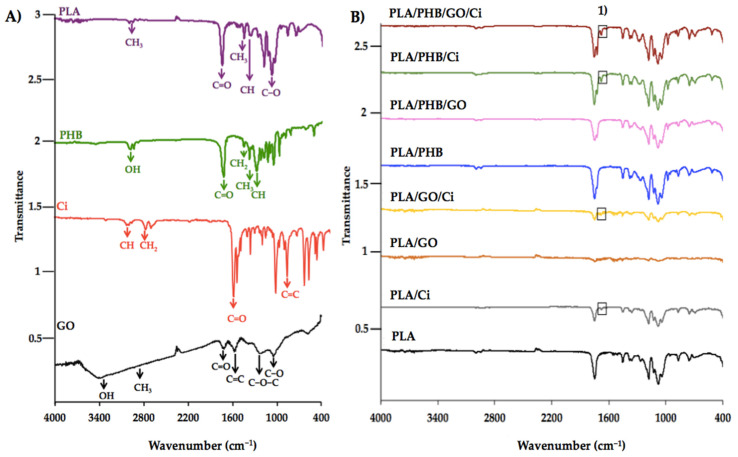
IR spectra of (**A**) PLA, PHB, Ci, GO and (**B**) blends and bionanocomposites. (**1**) Ci impregnation in films and blends.

Through the FTIR test, it was possible to identify the specific functional groups of polymers, GO and Ci, where the characteristic bands indicate the different vibrations and stretching associated with each group of the samples. In Figure 2A, PLA, PHB, Ci, and GO spectrums are presented, while in Figure 2B, those of the films and blends with the nano-reinforcement and active compound are shown.

In the case of GO (Figure 2A), a peak was observed around 3410 cm^−1^, 1723 cm^−1,^ and 1616 cm^−1^ associated with the stretching of the –OH, C=O, and C=C groups, respectively [56]. It was possible to detect a peak at 1224 cm^−1^ indicating the vibration of the –C–O– groups, meanwhile neat to 1050 cm^−1^ can be attributed to stretching of the epoxide C–O groups at 1051 cm^−1^ [57]. Also, Ci was analyzed by FTIR, and it is possible to observe there is a sharp peak at 1668 cm^−1^ indicating the presence of carbonyl uptake C=O, these structural carbonyl clusters are conjugated with double bonds. The peak at 2929 cm^−1^ refers to the CH_2_ group and the 3044 cm^−1^ to CH. The existence of an aromatic ring is indicated by the 1450 cm^−1^. The presence of a bond C=C indicates the region in the absorption of wave number 689 cm^−1^ [28,39].

On the other hand, characteristic peaks of the polymers used were identified. In the PLA case, peaks were observed around 2994 and 2945 cm^−1^ associated with the asymmetric and symmetric stretching of the –CH– bond of methyl –CH_3_. A peak associated with the carbonyl group C=O, characteristic of this polymer, can be seen at approximately 1748 cm^−1^ [26]. While the three peaks at 1180 cm^−1^, 1129 cm^−1^, and 1080 cm^−1^ are assigned to asymmetrical vibrations of C–O–C and C–O [58]. Lastly, peaks at 749 cm^−1^ and 870 cm^−1^ are assigned to the stretching of the C–C bond, attributed to the crystalline and amorphous PLA phases [59,60]. In the PHB case (Figure 2B(1)), there are two peaks 2967 and 2925 cm^−1^ associated with the presence of the alkyl –CH3 group [61]. It can also observe a peak at 1724 cm^−1^ belonging to the C=O ester bond, the stretching of –C–O–C– is possible to observe it in the peaks 1282 cm^−1^, 1230 cm^−1^, 1184 cm^−1^, 1133 cm^−1^, and 1101 cm^−1^. Finally, the peaks identified at 1724 cm^−1^, 1282 cm^−1^, and 1230 cm^−1^ are related to the crystalline phase of PHB [62]. There are differences in the state of the crystalline order between both polymers; PHB (<60%) is more crystalline than PLA (~5%) [63]. The characteristic peak of amorphous carbonyl vibration for PLA can be observed around 1748 cm^−1^, in agreement with the data published by Arrieta and coworkers (2017) [21]. While the carbonyl stretching of the PHB corresponding to the crystalline phase is at 1724 cm^−1^ and that of the amorphous phase at 1740 cm^−1^ [64].

On the other hand, the incorporation of GO in the PLA matrix produced a shift of the peaks from 1723 cm^−1^ to 1736 cm^−1^, this could be associated with the interaction with carbonyl groups. Davoodi and coworkers (2018) [26] indicated that when incorporating GO to PLA, they observed the appearance of peaks between 1611 cm^−1^ and 1623 cm^−1^, corresponding to the vibration of the C=C bonds in the structure of the graphene oxide layers. Meanwhile, for PLA/PHB/GO, a decrease in intensity in the band close to 1740 cm^−1^ was observed, evidencing an interaction between GO and the polymeric matrix. By contrast, for the PLA/PHB/GO/Ci film a displacement of this band was observed at 1752 cm^−1^, which suggests an interaction that generates a possible change in the matrix crystallization of the matrix. Finally, in Figure 2B(1) the appearance of the bands between 1600 cm^−1^ and 1700 cm^−1^ visualized in impregnated films—such as PLA/GO/Ci, PLA/PHB/Ci, and PLA/PHB/GO/Ci—are related to the vibration of the aromatic ring and Ci aldehyde group [28].

#### 3.1.3. Thermal Properties

Thermal properties of all materials were analyzed using a Differential Scanning Calorimeter Mettler Toledo DSC-822e and Thermogravimetric analysis (TGA) tests were performed with a Mettler Toledo Gas Controller GC20 Stare System TGA, both experiments were carried out under nitrogen atmosphere.

**Figure 3:**
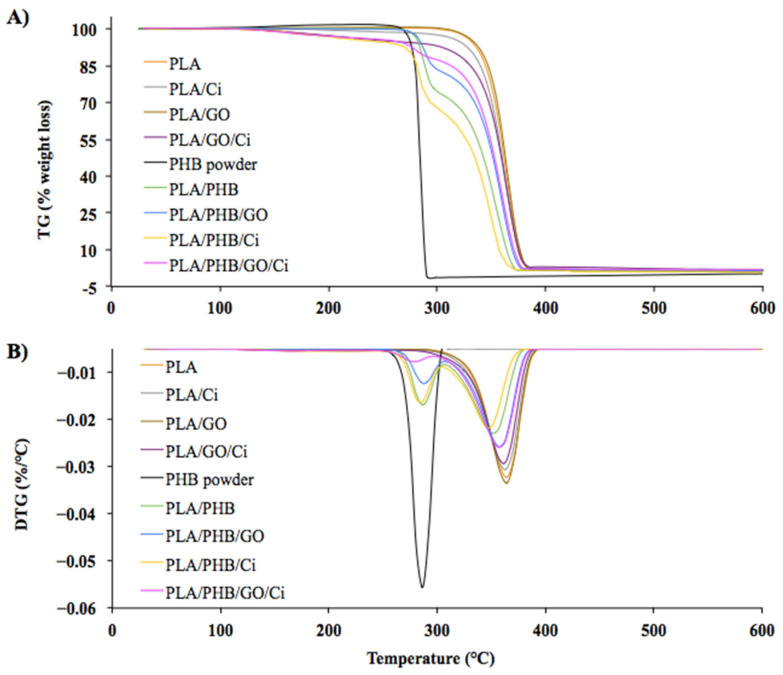
TG (**A**) and DTG (**B**) curves obtained for PLA, PHB, blends, and bionanocomposites.

As a result, the PLA and PHB powder film samples show a single degradation step (Figure 3A). For PLA film, this temperature was close to 365 °C, it is well-known that the thermal degradation of PLA is complex and various mechanisms can be associated with its degradation, such as the hydroxyl end-initiated ester interchange process and chain [65]. While the PHB powder presented a degradation temperature of 283 °C, which is caused almost exclusively by a random scission on the ester bond at high temperatures [66,67].

In the GO case, two inflection curves can be seen, where the first is between 42–120 °C and the second 175–243 °C. The first corresponds to the loss of absorbed moisture and the second is associated with the elimination of carbonyl, epoxy, and hydroxyl groups [26].

Due to its structure, GO has a potential action as an effective thermal barrier in the PLA matrix. The literature indicates that if there is a strong interfacial adhesion between the nanomaterial and the polymer, it could be possible to restrict the mobility of the chains by improving their thermal stability [68]. For this case, considering that there was a low peak displacement associated with the interaction between the carbonyl groups (see Figure 3B), it could be concluded that there is a lack of uniform dispersion or formation of aggregates, which allows stability in the degradation temperature [69]. For the case of the PLA/PHB blend (Figure 3B), two DTG were obtained between the first at 286.7 °C and the second at 350.8 °C, these values if compared with other studies of the same blend observing lower temperatures. This increase in the DTG can be explained due to a higher % *w*/*w* of PLA in the blend provoking higher thermal stability [21,70]. The low PHB thermal stability is due to the fact that it interacts with the amorphous phase of PLA through trans-esterification reactions [21], generating a negative effect on the stability of the materials in general. Considering this effect, when observing Figure 3B it is possible to appreciate two thermal degradation temperatures, associated with the respective polymers, showing that they are immiscible [70].

On the other hand, the GO in PLA/PHB/GO materials did not generate a variation on the DGT. Some authors indicate that the compounds derived from graphene have the advantage of being good electron acceptors, forming donor-acceptor complexes with the by-products of the nucleophilic chain cleavage of derivatives such as PHBV during their thermal degradation [71]. It is observed that the PLA/Ci also two curves DTG, the first step associated with Ci degradation, as reported in previous works [28,72] where degradation temperature was indicated at near 130 °C followed by the total PLA thermal degradation. Besides, the incorporation of the active compound by supercritical impregnation caused a decrease of 5 °C in the PLA/PHB/GO/Ci bionanocomposites and an increase of 5.4 °C with respect to the PLA/PHB/Ci film with respect to the neat PLA. Given this, it is associated with the fact that the Ci incorporation in a polymeric matrix with 0.5% *w*/*w* GO and PHB, manages to increase the range of thermal stability in the blend.

**Figure 4:**
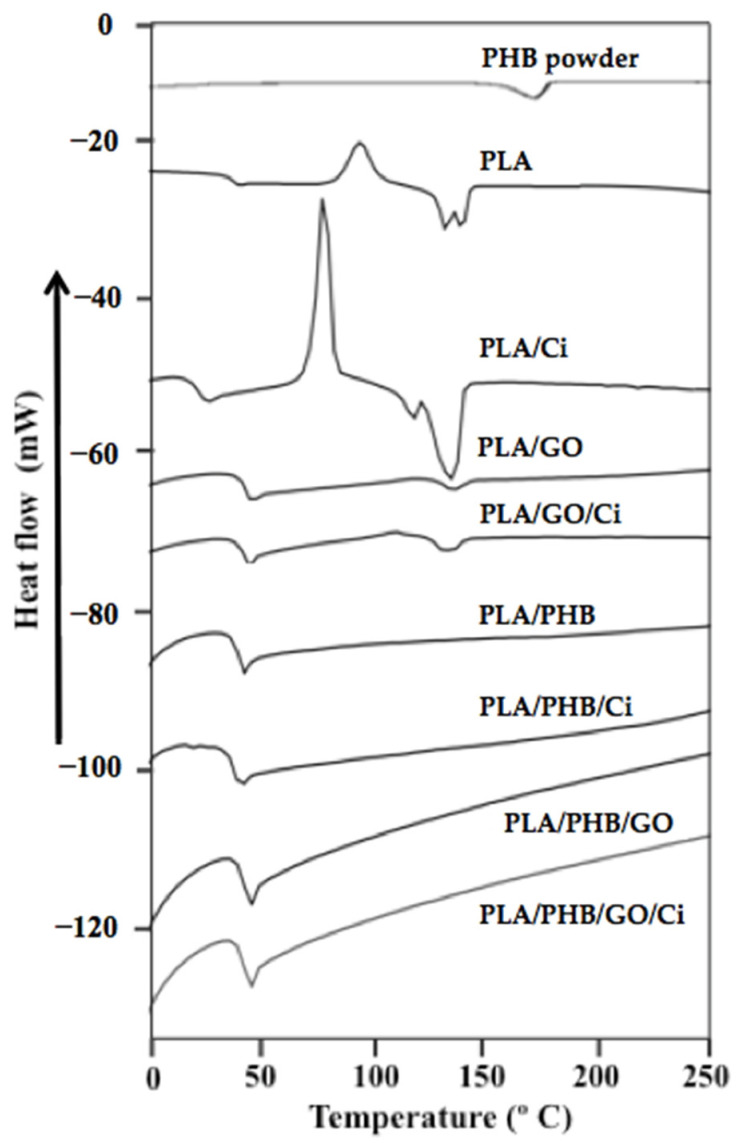
DSC thermograms of different materials obtained.

To evaluate the thermal properties and miscibility of the blends and bionanocomposites, a DSC analysis was carried out (Figure 4), thus evaluating the transition temperatures of the samples obtained. The curve of the second heating was calculated, from which the glass transition temperature (T_g_), crystallization temperature (T_cc_), melting temperature (T_m_), and the percentage of crystallinity (X_c_) were determined.

From Figure 4, it is possible to observe the thermogram of the high molecular weight PHB powder obtained from *Azotobacter vinelandii* bacteria, which presented a melting temperature (T_m_) ≈ 163 °C. This value was similar to that reported by Domínguez-Díaz and coworkers (2015), who studied the physicochemical differences of PHBs with different molecular weights obtained from the same bacteria. From this research, it is indicated that thermal properties are influenced by molecular weight and not production conditions. The significant decrease in T_m_ and crystallinity at very high molecular weights suggest that molecular entanglements may be playing a role in compromising crystallization and therefore the degree of crystallinity is smaller. That is, as crystallization during cooling from the melt requires macromolecular diffusion and rearrangement, the entangled molecular network would act as barriers to molecular motion and fewer chains would crystallize giving a weak crystallization exotherm. Once the molten to solid transition is completed some chains in the amorphous phase would be in a metastable state, hence when re-heating the sample these chains would have enough energy to crystallize, and consequently, a cold crystallization process is observed. In summary, PLA/PHB blends do not exhibit T_m_, and this behavior could be attributed to different causes. Firstly, the high molecular weight of PHB could cause an impediment to the formation of the crystal that will later be melted, showing a close correlation between the crystallinity reduction and the melting temperature. Secondly, it is also possible that the stereo-regularity of the PHB plays a role in the significant reduction of T_m_ and crystallinity degree [73] and thirdly, the GO could collaborate with the impediment of crystallization, in combination with the saturation of Ci miscibility. The values of thermal properties obtained from neat PLA correspond to that indicated in the literature [28,34,69] as well as those reported for the PLA/PHB blend [21,62,74].

On the other hand, it is possible to observe that the incorporation of PHB to the PLA matrix did not generate a significant effect on T_g_ value, obtaining temperatures of 49, 50.7, and 50 °C for PLA/PHB, PLA/PHB/GO, and PLA/PHB/GO/Ci, respectively. However, there is a 5 °C decrease when Ci was incorporated into the PLA/PHB blend, similar values were obtained in the same blend when 20% lactic acid oligomers were incorporated, causing a plasticizer effect associated with the compound incorporation [11].

Regarding T_cc_, Ci caused a decrease of 18.6 °C in the PLA matrix, this is due to the fact that the active compound promotes the formation of less stable α’ crystals, causing T_cc_ values below 100 °C [28]. The opposite effect caused GO an increasing T_cc_ ≈ 12 °C, this could be associated with a nucleation effect in places with greater dispersion [68]. The blends did not show T_cc_ due to the effect caused by the high molecular weight PHB and the increase in the amorphous phase influenced by the active compound presence. Regarding the crystallinity percentage (X_c_), the values for PLA (4%) and PLA/Ci (1.5%) were determined, whose magnitudes are in accordance with the reported in the bibliography [28].

On the other hand, for the PLA/GO/Ci it was obtained a crystallinity percentage (X_c_) is equal to 1.6%. From these results, it is inferred that the GO presence does not exert a significant effect, reiterating the effect on the reduction of chain movement [75].

#### 3.1.4. Mechanical Properties

Tensile properties such as Young’s Modulus (MPa), Tensile strength (MPa), and Elongation at break (%) are shown in Table 1 for all materials obtained. Young’s Modulus value for PLA is similar to that recorded in other investigations [21,28,34] when incorporating GO to the polymeric matrix PLA, a significant decrease in this parameter is observed.

Pinto and coworkers (2013) developed thin films of PLA with small amounts (0.2 to 1 *w*/*w* %) of graphene oxide (GO) and graphene nanoplatelets (GNP). When studying the mechanical properties they observed that with a GO content of 0.3 *w*/*w* %, Young’s modulus increases but for larger GO contents, the performance decreases probably due to less homogeneous distribution and agglomeration of GO particles within the PLA, which could also to attribute to the wrinkled morphology of GO which is less favorable for interactions with the polymer matrix. However, it would be expected that incorporation of GO would lead to a stronger nano-reinforcement effect than the incorporation of GNPs, considering that the presence of oxidized groups over the whole surface would favor interactions with hydrophilic groups of PLA [27]. In our case, the use of extrusion process and masterbatch method, there is the advantage of a much more economical and ecological large-scale elaboration process, however, the difficulty of achieving a homogeneous dispersion level is still present, due to the strong interactions of van der Waals between the sheets of the nano-reinforcement, facilitate the generation of agglomerations [76]. Regarding the films impregnated with the active compound, a significant decrease in the elasticity modulus can be observed in the materials. This behavior can be attributed to the plasticizing effect of Ci and scCO_2_ [28]. For the case PLA/PHB/Ci blend, it presented a value of 526 MPa. Compared to the PLA film this increase could be attributed to the recrystallization of the PLA. This phenomenon occurs in blends that present lower Tg values than the virgin polymer, for this case the PLA obtained a Tg of 50.9 °C while the PLA/PHB blend presented a Tg of 49 °C, this variation will depend on the degree of miscibility of the blende and storage conditions [68].

The tensile strength decreased significantly in the films and blends with the incorporation of Ci. This effect could be associated with the cohesion of the molecular chain when the active compound is incorporated, generating a loss of rigidity of the PLA, due to the fact that Ci interferes with the chain-to-chain interactions of the polymer, again generating the plasticizing effect Ci in PLA films [28] and PLA/PHB blends.

The cinnamaldehyde impregnation in PLA films causes a significant increase of elongation at break (%). This effect involves a positive increase in the ductility, which could be explained by the improvement of polymer chain mobility, and which is also related to the plasticizing effect of the activities incorporated in the polymeric matrix [39]. The GO did not cause a significant variation of this parameter with respect to PLA films; this could be due to a decrease in the mobility of the chains due to the action of the nano-reinforcement [55].

### 3.2. Disintegration under Composting Conditions

**Figure 5:**
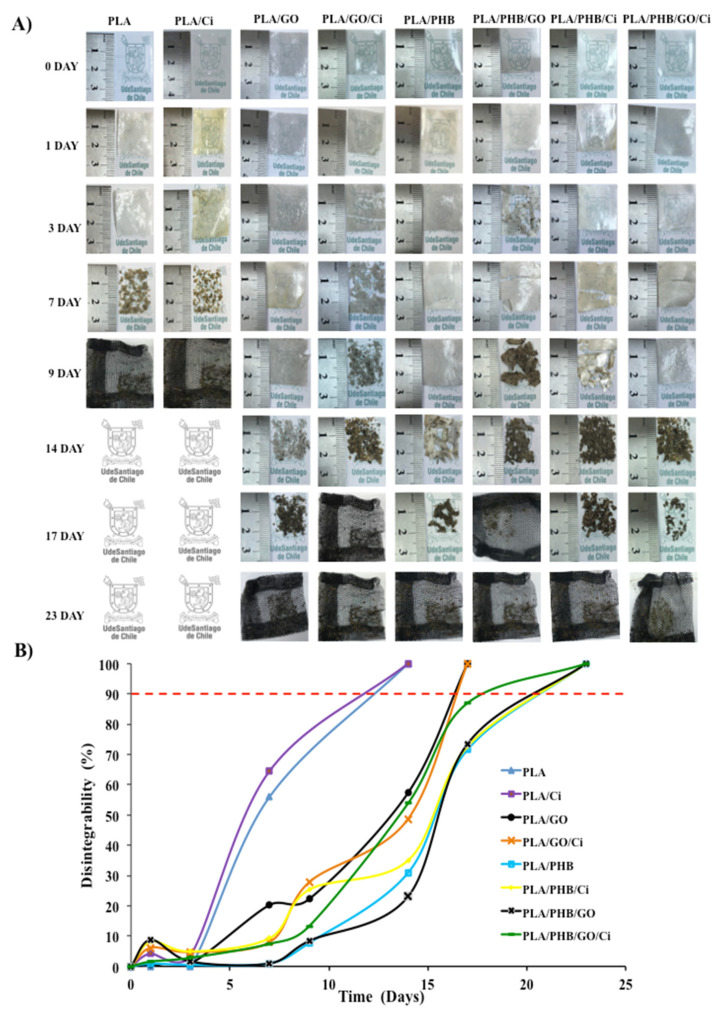
(**A**) Visual appearance of developed materials at different times under composting conditions, (**B**) Films disintegration degree under composting conditions as a time function.

Figure 5A shows the visual evolution of the disintegration of the blends and bionanocomposites during the 23 days that the test lasted. From the results, it is possible to confirm the biodisintegrable character of all formulations under composting. After 1 day of incubation from the beginning of the degradation process, PLA films’ loss of transparency and became more opaque. This behavior is because of a change in the refraction index of the materials as a result of water absorption and/or the presence of products formed by the hydrolytic process [77]. After three days of incubation in a composting medium, PLA/PHB/GO films became breakable due to the increase in their chemical degradation, where the PHB delayed the disintegration rate of PLA in the compost. This result is associated with the blend increasing the hydrophobic character, which delays the polymer hydrolysis process required for the microorganisms from the compost to further disintegrate the polymeric matrix. However, this conclusion may be affected by the film’s immiscibility, previously established by means of thermal analysis. Although these can present chemical interactions associated with band displacement (see Section 3.1), with respect to their chemical groups, the heterogeneity of the material influences their degradation. Given this, the particular role of PHB as a polymer matrix can be considered, since it has been proven that it delays the PLA degradability at a temperature of 58 °C, due to its different mechanisms at the time of degradation [70,78].

On the other hand, the disintegration process was accelerated in samples with impregnated Ci during the initial phases of the disintegration (7 days) because of their plasticizing effect, where the higher polymer chains mobility speeds up the hydrolytic disintegration process. Bubbles were observed in films with GO, related to the presence of water in the matrix, due to possible interactions with hydroxyl groups of the nano-reinforcement [79]. On the other hand, visual observations were confirmed by calculating the disintegration degree in terms of mass loss as a function of incubation time using the Boltzmann function to correlate the sigmoidal behavior of the mass loss during the disintegrability in the composting process (Figure 5B). In the figure, the line red at 90% of disintegration represents the goal of the disintegrability test. A clear increase of the disintegration phenomenon was observed in blends and bionanocomposites where the active Ci was impregnated during the initial phases of the disintegration due to their plasticization effect increasing the polymer chain mobility. Higher disintegration rates have been previously observed in PLA blended with essential oils such as D-limonene and also ascribed to its plasticization effect [80] and bionanocomposites with thymol and cinnamaldehyde impregnation [37]. This same behavior was obtained by evaluating the disintegration when incorporating carvacrol in films based on PLA/PHB plasticized with oligomeric lactic acid (OLA) and reinforced cellulose nanocrystals (CNC), where all formulations were disintegrated in less than 17 days [20]. The increased polymer chain mobility influences the diffusion process resulting in higher hydrolysis leading to small molecules (monomers and short-chain oligomers) that are available for the subsequent microorganisms attack [78]. Moreover, the disintegration rates are usually related to the surface wettability of materials. In this investigation, the rate of disintegration under composting conditions followed the same tendency of thermal and mechanical properties. The rate of disintegration under composting conditions was slightly longer with the presence of GO and in the blends, for example, PLA/GO presented a half-maximal degradation (t_50_) at 13.15 and PLA/PHB/GO showed t_50_ at about 15.59 days, with respect to neat PLA that showed t_50_ at about 6.48 days due to possible interactions with hydroxyl groups of the nano-reinforcement [81].

Given this, and as shown by the PLA/PHB/GO/Ci film, where GO and Ci are present, there was a significant increase showed t_50_ at about 13.52 days compared to the rest of bionanocomposites. This is associated with a synergistic effect in the PLA/PHB matrix, where the incorporation of Ci with scCO_2_ allows greater interaction between the matrix polymers, referring to their amorphous areas, allowing an increase of up to 16% in elongation to the break. This interaction was visualized by the FTIR test, reiterating what was indicated regarding the PLA band at 1750 cm^−1^ assigned to an amorphous stretch of the carbonyl group, as for PHB, with a band at 1748 cm^−1^, in relation to its amorphous state, as mentioned above [21,28], probably due to the effect of the use of the GO nano-reinforcement and the active compound Ci (plasticizer), simultaneously.

### 3.3. Release Kinetics of the Active Compound from Materials with and without Nano-Reinforcement

**Figure 6:**
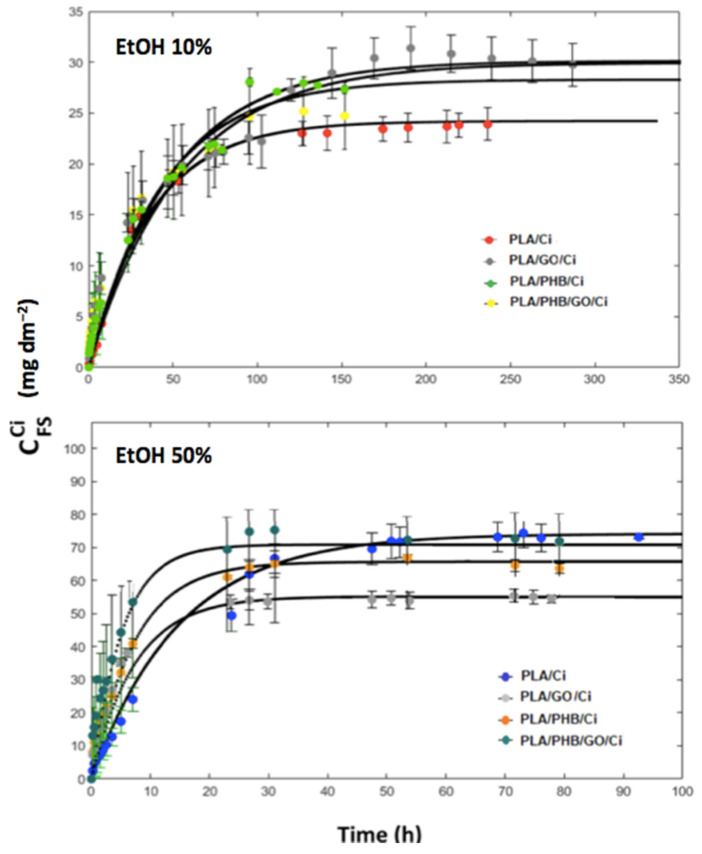
Release kinetics of cinnamaldehyde from the different polymeric systems into different solutions.

Cinnamaldehyde (Ci) release kinetics from PLA film, PLA/PHB blends, and the bionanocomposites containing graphene oxide (GO) at 0.5 *w*/*w* % (PLA/GO and PLA/PHB/GO) were characterized by means of experimental mass transfer assays with the aim to describe the transport and thermodynamics parameters active release process from the different polymeric structures. The experimental analysis was described in Section 2.7.1. The equilibrium conditions were expressed in terms of the partition coefficient of cinnamaldehyde (K_P/S_), which is a thermodynamic parameter that corresponds to the ratio between the Ci concentration in the polymer (P) and the simulant solution (SS). Meanwhile, the kinetic process was defined in terms of an effective diffusion coefficient (D_eff_) of Ci, which explains the rate of the active compound that diffuses through the polymeric structures. As shown by release kinetic curves in Figure 6, theoretical curves generated by the mass transfer model highly fitted with the experimental data. This fact indicated that the Ci release from the different polymeric systems based on PLA, PHB, and GO were mainly controlled by the diffusion phenomenon in the polymer structure defined by the second Fick’s law. The curve slope that represents the kinetics of the Ci concentration values in the solution greatly depended on the polymeric system analyzed and the solution in contact with the polymers.

Table 2 shows the partition coefficients (K_P/S_) of Ci for the different polymeric systems in both solutions. These values for each release assay were calculated by the quantification of the Ci amount remaining in the polymeric films by a solvent extraction procedure and HPLC determination. This thermodynamic parameter characterized the relative affinity of Ci towards the polymeric phase and the liquid solution. K_P/S_ values for each polymeric structure were dependent on the simulant solution and the lowest K_P/S_ values were obtained using EtOH 50% as Ci as the receiver phase. This effect of the EtOH content on the K_P/S_ has been previously reported for other naturally occurring compounds as thymol [34] and curcumin [82] and was related to the high chemical affinity of ethanol towards these compounds and to the PLA plasticization due to liquid simulant absorption during the release assays, which promotes the Ci mass transfer. In EtOH 50% solution, the K_P/S_ value for the PLA/Ci film (K_P/S_ = 135) was practically the same as the K_P/S_ value (K_P/FS_ = 133) reported by [31] for the distribution of Ci between impregnated electrospun PLA mats and EtOH 50% at 40 °C, even considering that the initial amount of Ci in the electrospun structure (3.29 *w*/*w* %) was lower than in the extruded PLA film used in our study (7.7 *w*/*w* %). This fact agrees with the rules established by thermodynamics for the phase equilibrium. For a nonreactive system fixing pressure, temperature, amount, and type of release simulant and polymer the degrees of freedom for the system becomes equal to 0 and the value for the distribution of a solute between the polymer and liquid solution must be a constant. The slight difference between the K_P/S_ values reported in our study and the reported by López de Dicastillo and coworkers (2018) could be the result of small differences in the mass of polymers used in the releases assays in each study [35].

As Table 2 shows, for EtOH, 10% receiving solution, the PLA/Ci films presented a K_P/S_ value (K_P/S_ = 950) at significantly lower for the PLA/PHB/Ci blend (K_P/S_ = 450), and bionanocomposites PLA/GO/Ci film (K_P/S_ = 460). The difference between the mono polymeric system (PLA) and the dual polymeric systems (PLA/PHB and PLA/GO) could be mainly explained by the different impregnations mechanism that governs the incorporation of Ci in each case [40]. In the mono polymer system (PLA) the Ci incorporation during the supercritical impregnation process could be mainly governed by the molecular dispersion mechanism instead of by the physical deposition mechanism as a consequence of the well-known high chemical affinity between Ci and PLA due to the hydrogen bond interactions between the oxygen of the aldehyde belonging to Ci and the hydrogen of the hydroxyl of PLA [28], which in turn generated a great cohesive force between PLA and Ci and decreased the amount of Ci able to transfer to the release simulant. On the contrary, the physical deposition could be the mechanism governing the incorporation of Ci in the dual polymeric systems as a consequence of the chemical interactions established between PLA, PHB, and GO during the extrusion process—which could have noticeably decreased the availability of carbonyl or end hydroxyl groups in the PLA structure able to interact with Ci. In this case, Ci remained mostly physically absorbed in PLA after the supercritical impregnation process, which in turn generated a low cohesive force between Ci and these structures, increasing the Ci amount able to migrate into the release solution. A synergic effect was observed when GO and PHB were added together to PLA, decreasing in more extent the affinity of Ci towards the polymeric phase and resulting in a higher amount of Ci migrated in the simulant. This effect was also significantly evidenced in the release assays done in EtOH 50% solutions (see Table 2).

Once K_P/S_ was experimentally obtained for each release assay, the D_eff_ of Ci in the different polymeric systems were correlated from the experimental data and the simulated specific migration assay applying the mass transfer model. Different values of diffusion coefficients of Ci in the polymeric systems were tested until finding the best agreement between the experimental release kinetics and theoretic data predicted by the mass transfer simulation model. Table 2 shows the D_eff_ of Ci through the different polymeric structures using EtOH 10% and 50% solutions as Ci receiving phases. Diffusion coefficients values of the active compound in all the polymeric systems were dependent on the type of solution. Using EtOH 10% as a liquid receiver phase, Ci presented a D_eff_ value in PLA/Ci (5.0 × 10^−15^ m^2^ s^−1^) slightly lower than the value calculated in the PLA/PHB/Ci blend (6.2 × 10^−15^ m^2^ s^−1^). This result was not expected because pristine PHB presents a high crystallinity and acts sometimes as a nucleating polymer agent, which should have resulted in a PLA/PHB blend with higher crystallinity and in turn with higher resistance to mass transfer than the neat PLA film. Nevertheless, PLA and PHB are not miscible in general [62], which is generated by extrusion of a non-cohesive polymeric blend film, which facilitates the diffusion of Ci during the release assays.

A higher increase in the D_eff_ of Ci (8.0 × 10^−15^ m^2^ s^−1^) was obtained for the PLA bionanocomposite film containing GO at 0.5 *w*/*w* % (PLA/GO/Ci). This effect could be related to irreversible changes in the physical properties of the nanocomposite due to the application of high-pressure CO_2_ as was reported for an LDPE nanocomposite film containing 5 wt% of the nanoclay Cloisite 20A [33]. Particularly, a greater increase in the free volume of the PLA/GO bionanocomposite with respect to the PLA/Ci film could have been obtained due to the poor compatibility between the polymer matrix and GO as was discussed in Section 3.1.1, which could have in this case promoted the diffusion of Ci during the release assay. Surprisingly, the diffusion coefficient of Ci (6.0 × 10^−15^ m^2^ s^−1^) was slightly decreased in the PLA/PHB/GO/Ci film, probably due to a better interaction between PHB and GO.

As Table 2 shows, when using EtOH 50% as a liquid receiving phase Ci presented in all the polymeric systems diffusion coefficients higher than the obtained using EtOH 10%, but following the same trend for the Ci diffusion coefficients; D_eff_ in PLA/Ci (4.7 × 10^−14^ m^2^ s^−1^) < D_eff_ in PLA/PHB/Ci (8.7 × 10^−14^ m^2^ s^−1^) < D_eff_ in PLA/GO/Ci (9.9 × 10^−14^ m^2^ s^−1^). The differences between receiving phases can be mainly explained by the swelling and plasticization due to the penetration of them inside the polymeric structures. These phenomena modified the effective transport properties of the polymers during the release assays, improving the mass transfer of Ci, and influencing the overestimation in the D_eff_ values in the polymer matrices.

The results and values of root mean square error (RMSE) of the model solution related to experimental data are reported in Table 2. A good fit between calculated and experimental data was obtained, with values ranging between 0.8–2.0%. Similar behavior has been reported in other works [32,34,36,50], showing that the active mass transfer in the blends and bionanocomposites is mainly controlled by the diffusion phenomenon in the polymer given by the second Fick’s law.

## 4. Conclusions

The impregnation of PLA/PHB blends and PLA/PHB/GO bionanocomposites with cinnamaldehyde by means of scCO_2_ was successfully achieved, obtaining results ranging between 5.7 and 6.1%. From a physical-chemical characterization of all developed materials, chemical interactions could be observed without noticeably showing an interaction between the nano-reinforcement and the active compound. Regarding the thermal properties of the developed materials, GO allowed to maintain the thermal stability of the PLA films, while the PHB polymer caused a decrease in its degradation temperature. Besides, with the GO and Ci incorporation, elongation percentage increased up to 16% obtaining more flexible materials, with respect to neat PLA.

The GO nano-reinforcing incorporation affected the diffusion rate of Ci in a 50% ethanolic solution, due to their possible agglomeration in the different polymeric matrices while the PHB did not significantly influence the Ci diffusion. All the materials exceeded the 90% disintegration capacity established by the ISO 20200 standard, although the GO and PHB presence slightly decreased the disintegration capacity of blends and bionanocomposites, reaching up to 17 and 23 days of testing, respectively. Finally, the results show that the extruded and supercritical developed bionanocomposites based on PLA/PHB blends could potentially be used for biomedical applications in the preparation of controlled drug delivery platforms, through the use of biodegradable patches for the management and wounds treatment, without compromising the biodegradability characteristics of the final material.

## Figures and Tables

**Table 1:** Mechanical properties for the different materials developed.

Materials	Tensile Strength (MPa)	Tensile Modulus (MPa)	Elongation of Break (%)
PLA	2677 ± 190 ^a^	50 ± 4 ^a^	4.7 ± 1.2 ^a^
PLA/Ci	119 ± 50 ^c^	20 ± 4 ^b^	12.5 ± 5 ^b^
PLA/GO	532 ± 248 ^b^	40 ± 4 ^c^	4.4 ± 0.2 ^a^
PLA/GO/Ci	111 ± 68 ^c^	26 ± 4 ^b^	5.3 ± 0.6 ^a^
PLA/PHB	850 ± 413 ^d^	34 ± 12 ^c^	12 ± 5 ^b^
PLA/PHB/Ci	526 ± 264 ^b^	57 ± 4 ^d^	4.6 ± 0.5 ^a^
PLA/PHB/GO	951 ± 366 ^d^	38 ± 9 ^c^	8 ± 5 ^a^
PLA/PHB/GO/Ci	302 ± 163 ^c^	24 ± 12 ^b^	16 ± 8 ^b^

Mean value (*n* = 3) ± SD. Parameters in columns denoted with the same letters (a–d) do not differ statistically at the level of confidence 0.05.

**Table 2:** Partition (K_P/S_) and diffusion coefficients (D_eff_) and RMSE values of cinnamaldehyde from PLA different polymeric systems impregnated using scCO_2_.

Simulant	Polymeric System	K_P/S_	D_eff_ (m^2^ s^−1^)	RMSE (%)
EtOH 10%	PLA/Ci	950 ± 31	5.0 × 10^−15^	1.5
	PLA/GO/Ci	450 ± 38	8.0 × 10^−15^	2.0
	PLA/PHB/Ci	460 ± 37	6.2 × 10^−15^	1.9
	PLA/PHB/GO/Ci	380 ± 36	6.0 × 10^−15^	2.4
EtOH 50%	PLA/Ci	135 ± 20	4.7 × 10^−14^	1.1
	PLA/GO/Ci	160 ± 19	9.9 × 10^−14^	0.8
	PLA/PHB/Ci	57 ± 11	8.7 × 10^−14^	0.9
	PLA/PHB/GO/Ci	24 ± 5	1.5 × 10^−13^	1.7

Mean value (*n* = 2) ± SD in the K_P/S_ parameter.

## Data Availability

Data sharing not applicable.
